# Pilot study comparing the childhood arthritis and rheumatology research alliance consensus treatment plans for induction therapy of juvenile proliferative lupus nephritis

**DOI:** 10.1186/s12969-018-0279-0

**Published:** 2018-10-22

**Authors:** Jennifer C Cooper, Kelly Rouster-Stevens, Tracey B Wright, Joyce J Hsu, Marisa S Klein-Gitelman, Stacy P Ardoin, Laura E Schanberg, Hermine I Brunner, B Anne Eberhard, Linda Wagner-Weiner, Jay Mehta, Kathleen Haines, Deborah K McCurdy, Thomas A Phillips, Zhen Huang, Emily von Scheven, for the CARRA registry investigators

**Affiliations:** 1 University of California, San Francisco, 550 16th Street, 5th Floor, San Francisco, CA 94158 USA; 2 Emory University School of Medicine/Children’s Healthcare of Atlanta, 2015 Uppergate Dr, Atlanta, GA 30322 USA; 3 Texas Scottish Rite Children’s Hospital, 5323 Harry Hines Blvd, Dallas, TX 75390 USA; 4 Stanford University, 725 Welch Rd, Palo Alto, CA 94304 USA; 5 Ann & Robert H. Lurie Children’s Hospital of Chicago, 225 E. Chicago Ave, Chicago, IL 60611 USA; 6 Ohio State University College of Medicine, 480 Medical Center Dr. S-2056, Columbus, OH 43210 USA; 7 Duke University Medical Center, 2100 Erwin Rd, Durham, NC 27705 USA; 8 Cincinnati Children’s Hospital Medical Center, 3333 Burnet Ave, Cincinnati, OH 45229 USA; 9 Cohen Children’s Hospital Medical Center, 1991 Marcus Ave, Lake Success, NY 11042 USA; 10 University of Chicago Hospitals, 5841 S. Maryland Ave, MC 5044, Chicago, IL 60637 USA; 11 Children’s Hospital at Montefiore/Albert Einstein College of Medicine, 111 E 210th St, Bronx, NY 10467 USA; 12 Hackensack University Medical Center, 30 Prospect Ave, Hackensack, NJ 07601 USA; 13 University of California, 200 UCLA Medical Plaza, Los Angeles, 90095 CA USA; 14 Duke University Medical Center, 2400 Pratt, St. Durham, NC 27705 USA

**Keywords:** Juvenile systemic lupus erythematosus, Lupus nephritis, Cyclophosphamide, Mycophenolate, Corticosteroids, Consensus

## Abstract

**Background:**

To reduce treatment variability and facilitate comparative effectiveness studies, the Childhood Arthritis and Rheumatology Research Alliance (CARRA) published consensus treatment plans (CTPs) including one for juvenile proliferative lupus nephritis (LN). Induction immunosuppression CTPs outline treatment with either monthly intravenous (IV) cyclophosphamide (CYC) or mycophenolate mofetil (MMF) in conjunction with one of three corticosteroid (steroid) CTPs: primarily oral, primarily IV or mixed oral/IV. The acceptability and in-practice use of these CTPs are unknown. Therefore, the primary aims of the pilot study were to demonstrate feasibility of adhering to the LN CTPs and delineate barriers to implementation in clinical care in the US. Further, we aimed to explore the safety and effectiveness of the treatments for induction therapy.

**Methods:**

Forty-one patients were enrolled from 10 CARRA sites. Patients had new-onset biopsy proven ISN/RPS class III or IV proliferative LN, were starting induction therapy with MMF or IV CYC and high-dose steroids and were followed for up to 24 months. Routine clinical data were collected at each visit. Provider reasons for CTP selection were assessed at baseline. Adherence to the CTPs was evaluated by provider survey and medication logs. Complete and partial renal responses were reported at 6 months.

**Results:**

The majority of patients were female (83%) with a mean age of 14.7 years, SD 2.8. CYC was used more commonly than MMF for patients with ISN/RPS class IV LN (vs. class III), those who had hematuria, and those with adherence concerns. Overall adherence to the immunosuppression induction CTPs was acceptable with a majority of patients receiving the target MMF (86%) or CYC (63%) dose. However, adherence to the steroid CTPs was poor (37%) with large variability in dosing. Renal response endpoints were exploratory and did not show a significant difference between CYC and MMF.

**Conclusions:**

Overall, the immunosuppression CTPs were followed as intended in the majority of patients however, adherence to the steroid CTPs was poor indicating revision is necessary. In addition, our pilot study revealed several sources of treatment selection bias that will need to be addressed in for future comparative effectiveness research.

**Electronic supplementary material:**

The online version of this article (10.1186/s12969-018-0279-0) contains supplementary material, which is available to authorized users.

## Background

Systemic lupus erythematous (SLE) is a chronic and complex autoimmune disease which causes systemic inflammation and may involve any part of the body. Individuals diagnosed in childhood or adolescence have more aggressive disease compared to adults, with lupus nephritis (LN) occurring in up to 80% of children [1]. The diagnosis of LN is established by kidney biopsy and classified according to the 2004 International Society for Nephrology/Renal Pathology Society (ISN/RPS) criteria [2]. Proliferative lesions are classified as class III if the lesion is focal or class IV if the lesion is diffuse, involving over half the sampled glomeruli. Treatment usually involves six months of aggressive induction immunosuppression to induce renal remission, followed by years of maintenance immunosuppression aimed at preventing disease flares. Progression to end-stage renal disease may occur despite therapy with class IV LN patients at the greatest risk, estimated at 44% over 15 years [3].

Data demonstrating optimal therapy for proliferative LN in children and adolescents are lacking. Thus, there is significant variability in the treatment of children and adolescents with LN as providers rely upon extrapolation from adult SLE studies, pediatric renal transplant literature, limited retrospective studies, and anecdotal experience to guide medical decision-making [4]. In an effort to reduce treatment variability and facilitate comparative effectiveness studies in pediatric rheumatic diseases, Childhood Arthritis and Rheumatology Research Alliance (CARRA) developed Consensus Treatment Plans (CTPs) for several pediatric diseases, including one for proliferative LN [4]. However, there is little information about the acceptability and in-practice use of these CTPs.

Therefore, the primary aim of the pilot study was to demonstrate feasibility of the LN CTPs in terms of adherence to the treatment regimens and to delineate barriers to implementation (reasons for not following the CTPs) in clinical care in the United States. Further, we aimed to explore the safety and effectiveness of the treatments rendered upon completion of induction therapy.

## Methods

### Consensus treatment plans of LN

Details about these plans have been previously published [4]. In brief, for induction therapy of proliferative LN, CARRA CTPs recommend either intravenous (IV) cyclophosphamide (CYC) 500–1000 mg/m2 (max 1500 mg) every 4 weeks × 6 months (6–7 doses) or mycophenolate mofetil (MMF) 600 mg/m2/dose BID (ma× 3000 mg/day) in addition to one of three high-dose corticosteroid (steroid) CTPs. Steroid CTP options include primarily oral, primarily IV, or mixed oral/IV regimens. High-dose pulse IV methylprednisolone 30 mg/kg (max 1000 mg/dose) × 3 doses is recommended at the start of therapy in the primarily IV and mixed oral/IV steroid CTPs and optional in the primarily oral steroid CTP. Tapering schedules for prednisone or prednisolone are outlined for each steroid regimen. Use of mesna, anti-emetics, gonadotropin releasing hormone agonists, and antimicrobials for *Pneumocystis jiroveci* prophylaxis are at the provider’s discretion. Maintenance immunosuppression CTP options include MMF, azathioprine (AZA), or quarterly IV CYC in addition to low-dose prednisone or prednisolone with a goal to taper to ≤10 mg/day by 12 months and to ≤5 mg/day by 24 months from the start of induction therapy.

### Study design and patient population

A multicenter prospective observational cohort study was conducted from May 2012 through October 2015. Patients at participating sites were enrolled in the CARRA registry and treated per the induction CTPs at the discretion of the pediatric rheumatology provider. Patients with complete or partial renal response at the 6-month visit were treated according to one of the three maintenance CTPs. Main study entry criteria included new diagnosis of biopsy-proven active proliferative LN (ISN-RPS class III or IV) with or without concurrent class V disease, fulfillment of ≥4 of 11 American College of Rheumatology revised classification criteria for SLE or presence of 3 criteria provided one is histological evidence of LN [5], age at diagnosis with SLE ≤ 16 years, and age at study enrollment ≤20 years. Exclusion criteria were: severe infection, pregnancy or lactation, presence of another chronic or genetic disease or organ involvement that significantly influenced treatment of LN, and treatment with MMF or CYC not indicated per provider.

### Data collection

Study visits occurred at baseline and 3, 6, 9, 12, 18, and 24 months from the start of induction therapy. Standard-of-care clinical and laboratory data were captured at each visit. Data was collected using standardized case report forms through the InForm electronic data capture system managed by the Duke Clinical Research Institute. Patients or guardians were consented for data collection through the Legacy CARRA registry. The Legacy CARRA registry general protocol and consent was approved by Duke University institutional review board (IRB) and all participating site IRBs. Because the CTP study is not interventional and patients receive standard-of-care treatment at the discretion of their provider, only consent for data collection as a participant in the CARRA registry was required.

### Reasons for CTP selection

The provider’s reasons for CTP selection were assessed using standardized responses (Table 1) with the ability to select multiple reasons. Reasons for induction immunosuppression and steroid CTP selection were assessed separately at baseline. Reasons for maintenance immunosuppression CTP selection were assessed in responders at the 6-month visit.

**Table 1:** Standardized responses used to assess reasons for consensus treatment plan (CTP) selection

Induction and maintenance immunosuppression CTPs	Steroid CTPs
This is what I or my group always does	This is what I or my group always does
This treatment works best	I think this steroid regimen works best
This treatment is safer	I think this steroid regimen is safer
This treatment is better tolerated	I am concerned about my patient’s adherence with oral meds
I am concerned about my patient’s adherence with oral meds	My patient prefers oral meds
This is the only option covered by my patient’s insurance	Other (free text)
My patient prefers this method of medication administration	
My patient is very concerned about side effects	
Other (free text)	

### Feasibility of LN CTPs

Adherence to induction immunosuppression and steroid CTP regimens was assessed by medication log and provider-report. Medications used during the study period were recorded at every visit. Overall adherence to the induction CTPs was evaluated by providers at the 3- and 6-month visits by asking whether the CTP had been followed as intended. Reasons for not following a CTP were assessed by multiple choice with ability to select multiple reasons: patient non-adherence, patient-reported intolerance, physician drug adjustment due to intolerance, adverse event, disease flare, lack of response, laboratory abnormality, pregnancy, and other.

### Renal response

Renal response was assessed by providers at the 6-month visit. Responder criteria were established as part of the previously published CARRA LN CTP and adapted from the 2006 ACR response criteria for proliferative and membranous renal disease in SLE clinical trials [4, 6]. Complete renal response (CR) was defined as normalization of estimated glomerular filtration rate (GFR), inactive urine sediment (< 5 WBC/hpf, < 5 RBC/hpf, and no cellular casts) and spot urine protein-to-creatinine ratio (UPCR) < 0.2 mg/mg. Partial renal response (PR) was defined as at least 50% improvement in two core renal parameters (GFR, urinary sediment, proteinuria), maximum UPCR of < 1.0 mg/mg, and no clinically relevant worsening of the remaining renal core parameters. Laboratory measures of renal function were collected at every visit. GFR was estimated using the modified Schwartz formula [7].

### Disease activity

The Systemic Lupus Erythematosus Disease Activity Index 2000 (SLEDAI-2 K) score was reported at baseline and at each follow-up visit [8]. Providers assessed whether the patient had experienced a disease flare since the previous visit and whether the flare was renal or non-renal. Specific flare criteria were not provided.

### Safety

Adverse Events (AEs) were graded using the Common Terminology Criteria for Adverse Events (CTCAE v4.0) [9]. AEs of grade two and higher and serious adverse events were recorded at each study visit. Serious AEs were defined as death, life-threatening, hospitalization, disability or permanent damage, congenital anomaly or birth defect, or event that does not fit the defined outcomes but may require intervention to prevent one of the defined outcomes.

### Statistical analyses

This was not a randomized study and comparisons of baseline characteristics between CTP groups were performed using Chi-square test, Fisher’s exact test, and Wilcoxon rank sum tests to evaluate for possible biases impacting CTP selection. To quantify deviation from the oral steroid tapers during induction therapy, the difference between the expected dose recommended per the chosen CTP and the reported daily dose was calculated for each patient. An average percent daily difference for each week of induction therapy was generated. Deviations from the IV pulse component of the primarily IV and mixed steroid CTPs were calculated similarly by taking the difference between the expected (per CTP) and recorded number of pulses in the medication log.

Exploratory analyses on clinical outcomes were performed using multivariate logistic regression and mixed effect models for repeated measures. The impact of induction immunosuppression treatment (CTP) on the renal response at the 6-month visit was evaluated by multivariate logistic regression analyses with adjustment of baseline characteristics including age (years), proteinuria (mg/dL), class of proliferative LN (III, IV), steroid CTP regimen (primarily IV, mixed IV/oral, primarily oral). Colinearity of continuous covariates were examined. Differences in longitudinal outcomes of GFR, proteinuria, and SLEDAI-2 K between induction immunosuppression CTPs were assessed using mixed models with repeated measures with adjustment for baseline characteristics including age, gender, time of scheduled visits, steroid CTP regimen, and baseline values of these outcomes. Study treatment (CTP) was considered a fixed effect and subjects were considered random effects. Missing data points were considered missing at random. Multiple variance structures were explored such as unstructured and spatial power. If convergence was reached with multiple covariance structures, standard goodness-of-fit measures were used to select the model with the best fit. Statistical analyses were conducted using STATA® 14.0 (StataCorp LLC) and SAS® 9.3 (SAS Institute Inc.). All tests are two-sided. *P*-values were not adjusted for multiple comparisons. Tests with *p*-values of < 0.05 were considered statistically significant.

## Results

### Patients

Eighty-five patients were screened with 41 patients ultimately enrolled at 10 CARRA sites. The most common reasons for not participating were failure to meet inclusion criteria (66%) and provider decision not to use a CTP to guide treatment (20%). Baseline demographic and clinical characteristics are shown in Table 2. Significantly more patients in the CYC group had class IV LN (79% vs. 35%, *p* = 0.005) and hematuria (96% vs. 47%, *p* = 0.001) compared to the MMF group.

**Table 2:** Baseline characteristics overall and by induction immunosuppression CTP

	All *n* = 41	CYC *n* = 24	MMF *n* = 17	p-value
Demographics				
Age at enrollment in years, mean (SD)	14.7 (2.8)	15.2 (2.9)	14 (2.6)	0.146
Age at SLE diagnosis in years, mean (SD)	13.8 (2.8)	13.8 (2.9)	13.7 (2.7)	0.832
SLE duration in weeks, median (IQR)	6 (1–73)	14 (3, 120)	5 (1, 10)	0.130
Female, n (%)	34 (83)	18 (75)	16 (94)	0.109
Race, n (%)				0.889
White	16 (39)	10 (42)	6 (35)	
Black or African American	11 (27)	6 (25)	5 (29)	
Asian or Pacific Islander	6 (15)	4 (17)	2 (12)	
Other	8 (19)	4 (17)	4 (24)	
Hispanic ethnicity, n (%)	11 (27)	6 (25)	5 (29)	0.753
Parental Income in US $/year, n/total (%)				0.798
< 25,000	6/26 (23)	4/15 (27)	2/11 (18)	
25–49,999	8/26 (31)	3/15 (20)	5/11 (45)	
50–74,999	2/26 (8)	1/15 (7)	1/11 (9)	
75–99,999	6/26 (23)	4/15 (27)	2/11 (18)	
100–150,000	2/26 (8)	2/15 (13)	0/11	
> 150,000	2/26 (8)	1/15 (7)	1/11 (9)	
Insured, n (%)	36 (88)	20 (83)	16 (94)	0.382
Clinical and laboratory characteristics				
Lupus nephritis class, n (%)				0.005
ISN-RPS Class III, n (%)	16 (39)	5 (21)	11 (65)	
ISN-RPS Class IV, n (%)	25 (61)	19 (79)	6 (35)	
Concurrent ISN-RPS Class V LN, n (%)	14 (34)	6 (25)	8 (47)	0.142
GFR in ml/min/1.73 m2, median (IQR)^a^	94 (70–107)	93 (79–107)	95 (67–123)	0.864
Proteinuria in mg pr/mg cr, median (IQR)^b^	1.9 (1.1–4.7)	1.9 (1.3–4.6)	1.8 (0.8–4.7)	0.554
Hematuria present, n/total (%)	29/40 (73)	22/23 (96)	7/15 (47)	0.001
Hypertension, n/total (%)^c^	23/37 (62)	13/20 (65)	10/17 (59)	0.699
ESR in mm/hr., mean (SD)	50 (33)	44 (27)	60 (41)	0.304
Complement factor 3, median (IQR)	51 (39–75)	51 (39–71)	55 (31–98)	0.685
Complement factor 4, median (IQR)	6 (4–8)	6 (3–8)	6 (4–14)	0.578
Elevated dsDNA, n/total (%)	32/36 (89)	20/22 (91)	12/14 (86)	0.629
Antiphospholipid antibody present, n/total (%)	24/40 (60)	14/24 (58)	10/16 (63)	0.792
SLEDAI-2 K, median (IQR)	12 (8–20)	16 (10–20)	12 (6–22)	0.458
PGA, scale 0–10, median (IQR)	5 (3–6)	5 (3–6)	6 (2–6)	0.707
SLICC Damage Index, n/total (%)				0.677
Total Score 0	30/37 (81)	17/22 (77)	13/15 (87)	
Total Score 1	7/37 (19)	5/22 (23)	2/15 (13)	

*Abbreviations*: *CTP* consensus treatment plan, *dsDNA* double stranded DNA antibody, *CR* creatinine, *GFR* glomerular filtration rate, *IQR* interquartile range, *ISN-RPS* International Society of Nephrology-Renal Pathology Society, *IQR* interquartile range, *PGA* Physician’s global disease activity, *PR* protein, *SD* standard deviation, *SLEDAI-2 K* systemic lupus erythematosus disease activity index-2000, *SLICC* systemic lupus international collaborating clinics

^a^GFR estimated using modified Schwartz equation

^b^Proteinuria assessed by spot urine protein to creatinine ratio

^c^Hypertension defined as systolic or diastolic blood pressure ≥ 90th percentile, [21]

### Study retention and visit timeliness

All patients completed at least 6 months of follow-up. Retention declined over time with 35 (85%) and 18 (44%) patients completing the 12- and 24-month visits respectively. Overall, 60% of visits occurred within four weeks before or after the target visit date.

### Induction CTP selection

**Fig. 1:**
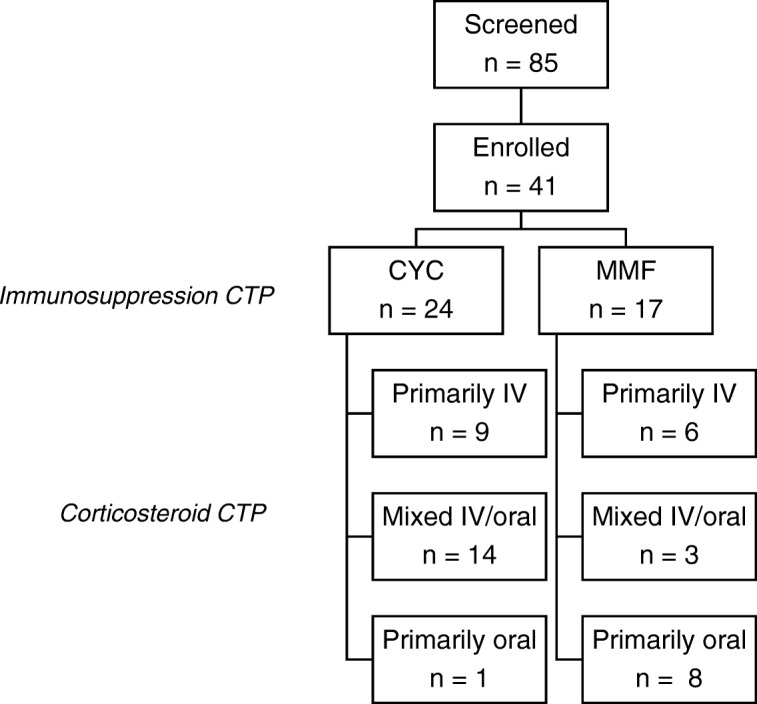
Enrollment and induction CTP selection. Abbreviations: CTP = consensus treatment plan, CYC = cyclophosphamide, IV = intravenous, MMF = mycophenolate mofetil, IV = intravenous

**Fig. 2:**
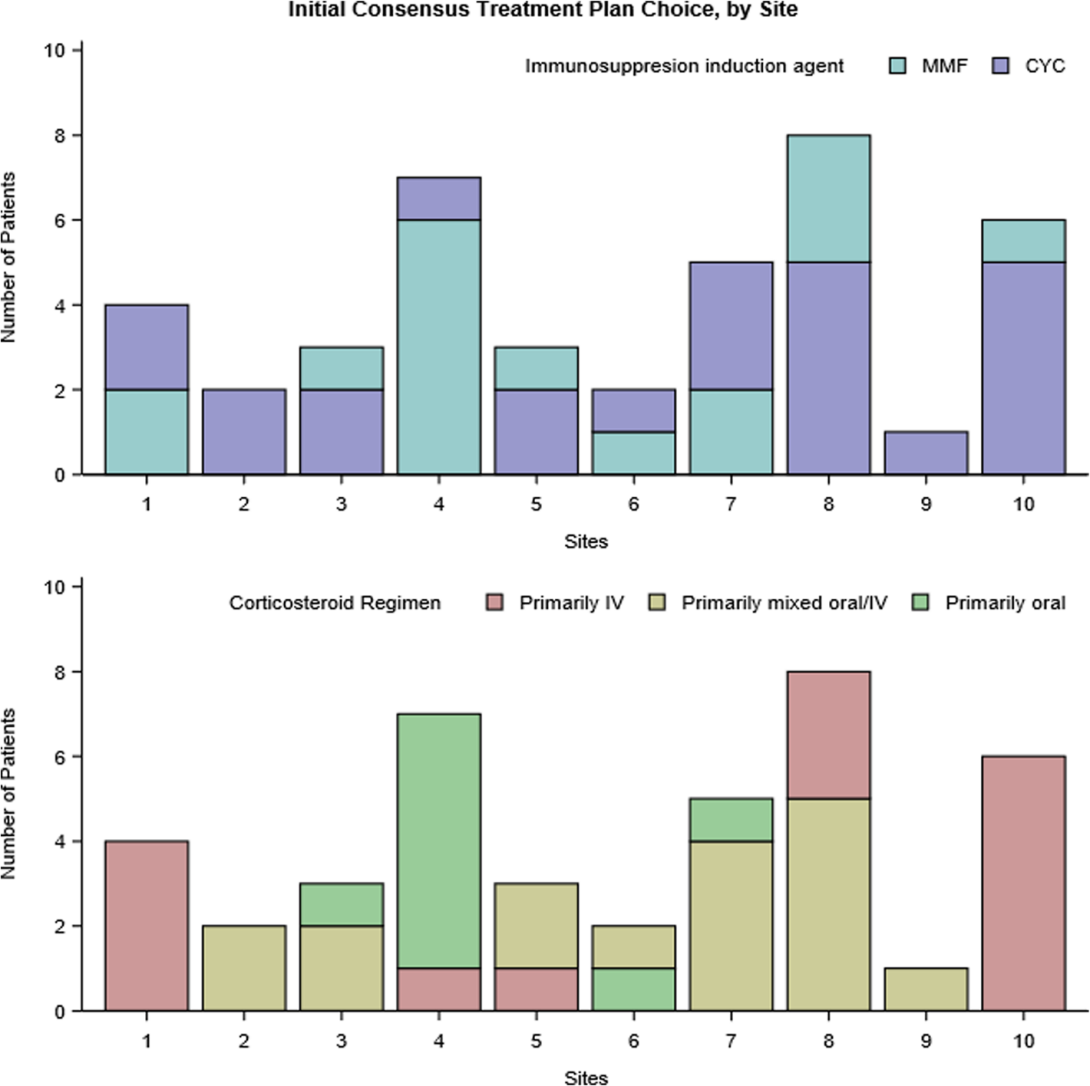
Induction CTP selection by study site. Abbreviations: CTP = consensus treatment plan, CYC = cyclophosphamide, MMF = mycophenolate mofetil

CYC was selected for 24 (59%) patients and MMF for 17 (41%) patients (Fig. 1). Most sites used both regimens (Fig. 2). The most common reasons for selecting CYC were “This is what I or my group always does” (54%) and “I think this treatment works best” (54%). Concern for patient non-adherence was the rationale for initiating CYC for 8 patients. The most common reason for MMF selection was “This is what I or my group always does” (41%).

Of the three steroid CTPs, the mixed regimen was the most commonly used (*n* = 17, 41%), followed by primarily IV (*n* = 15, 37%), and primarily oral (*n* = 22%). Several sites used only one regimen (Fig. 2). The most common reasons for CTP selection were: “I always select this regimen” (47%) and “This steroid regimen works best” (47%) for the mixed group, “This steroid regimen works best” (80%) for the primarily IV group, and “My patient prefers oral medications” (33%) for the primarily oral group. IV-based steroid CTPs (primarily IV and mixed) were more frequently used in conjunction with CYC (*p* = 0.002).

### Adherence to induction immunosuppression CTPs

Per medication logs, adherence to the immunosuppression CTPs was acceptable. In the MMF group, 84% and 86% of patients were at the target dose of ≥600 mg/m2 BID by the 3- and 6-month visits respectively, In the CYC group, 63% received the expected number of 6 or 7 infusions; the median number of infusions was 6 (IQR 5–6). The median cumulative CYC dose was 6290 mg (IQR 5040-8700). The median number of infusions was 6 (IQR 5–6) with a median monthly dose of 1100 mg/m2 (IQR 849–1256).

**Fig. 3:**
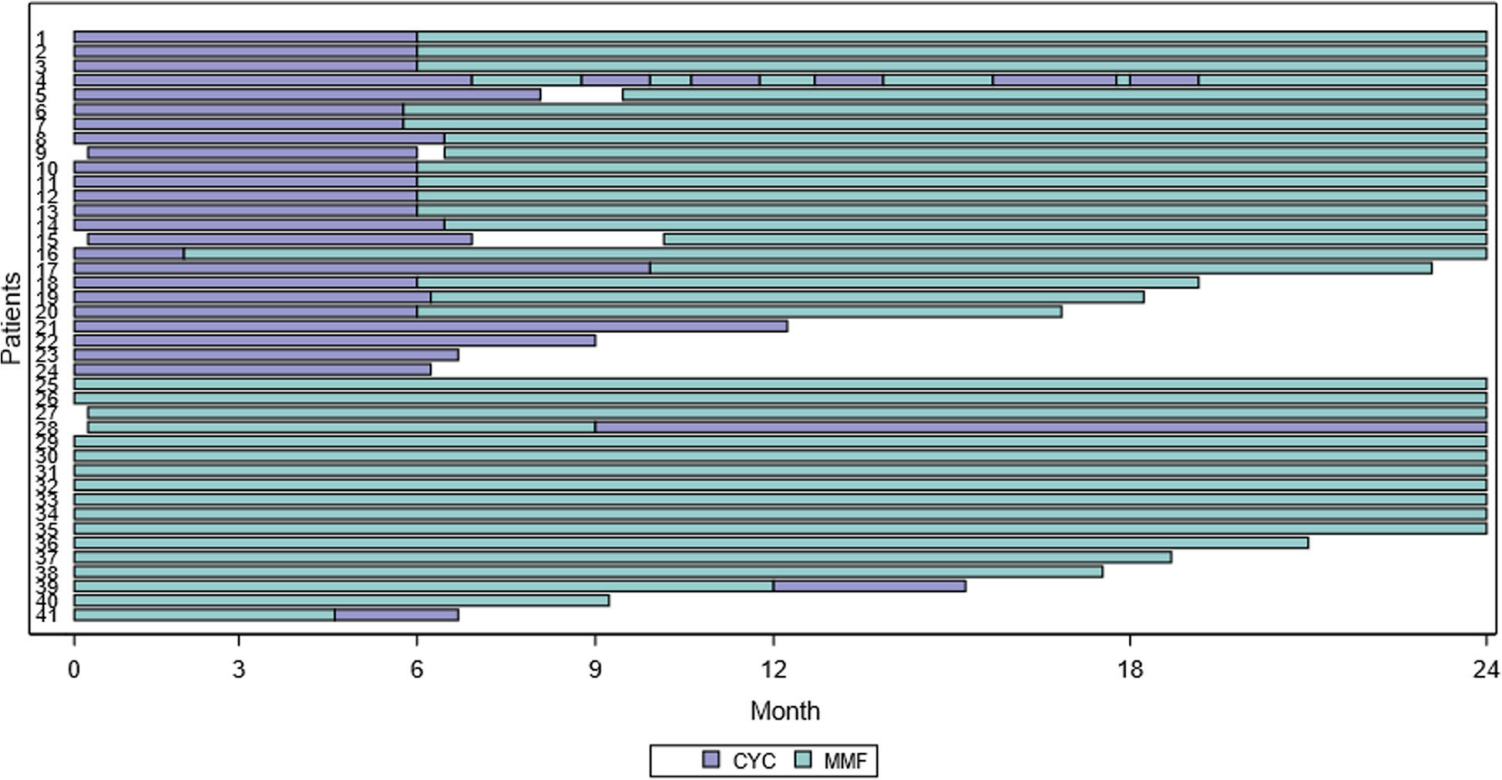
Pattern of CYC and MMF use and duration of follow-up. Abbreviations: CYC = cyclophosphamide, MMF = mycophenolate mofetil

Providers reported the immunosuppression CTPs were followed as intended in 76% of patients at the 3-month visit and 64% at the 6-month visit. The most common reason for not following a CTP as intended was patient non-adherence (17%). Although many providers reported not following a CTP, the vast majority (95%) of patients stayed on the initially selected treatment during the first 6 months (Fig. 3). Two patients switched therapy; one switched to MMF after the first CYC infusion due to an allergic reaction and another switched to CYC from MMF due to patient non-adherence. Two patients were treated with additional immunosuppression during the induction period. Concurrent medications are described in Additional file 1.

### Adherence to induction steroid CTPs

Oral steroid and IV pulse exposure through week 24 was highly variable, indicating poor adherence to the steroid CTPs (Table 3). For the primarily IV and mixed groups, there was a tendency to prescribe fewer IV pulses than outlined in the CTPs however, a substantial number of patients (*n* = 22) had incomplete IV records and were excluded from the IV analysis.

**Table 3:** Induction corticosteroid exposure through week 24 by CTP*

Steroid	Primarily IV *N* = 15	Mixed Oral/IV *N* = 17	Primarily Oral *N* = 9
Oral prednisone or prednisolone			
n patients with complete med log^a^	14	14	9
Total gm	5.8 (4–8.4)	8.4 (6.2–9.2)	16.1 (7.7–17.7)
Total mg/kg	119 (72–142)	144 (94–160)	285 (114, 313)
Difference from expected, mg^b^	− 108 (− 685, + 65)	+ 367 (− 692, + 1440)	− 338 (− 2650, + 1620)
IV methylprednisolone pulses			
n patient with complete med logs^a^	9	9	5
Total number of pulse doses	12 (6–14)	5 (3–8)	1 (0, 3)
Difference from expected, number of pulses^b^	− 1 (− 7, 0)	−5 (− 5, 0)	0 (0, 0)^c^

*All values presented as median (interquartile range), Abbreviations: CTP = Consensus treatment plan, IV = intravenous

^a^Patients with incomplete steroid records were excluded from analysis

^b^Difference from expected per CTP. A positive value (+) indicates more steroid was given than recommended per the CTP. A negative value (−) indicates less steroid was given per the CTP

^c^IV pulses optional in the primarily oral CTP

Providers reported adhering to the steroid CTPs in 68% of patients at 3 months and just 37% of patients at 6 months. Reasons for not following a steroid CTP were similar across the regimens; the most common reasons were patient non-adherence (22%) and other (17%). Review of free-text responses revealed a theme of tapering steroids more quickly than recommended.

### Maintenance CTP selection and steroid use

Patients with CR or PR at the month 6 transitioned to a maintenance CTP (*n* = 30). Twenty-eight patients (93%) were treated with MMF, two (7%) with quarterly CYC, and none with AZA. The most common reasons for selecting MMF were “This is what I or my group always does” (54%) and “I think this treatment works best” (54%).

The median prednisone or prednisolone dose at 24 weeks for responders was 12 mg/day (IQR 10–20) or 0.2 mg/kg (IQR 0.2–0.3). Of patients with complete tapering data at 12 months, 74% were in alignment with the CTP tapering goal of ≤10 mg/day (median 7.7 mg/day or 0.2 mg/kg/day, IQR 0.1–0.2). By 24 months, 78% were on a dose of ≤5 mg/day (median 3.4 mg/day or 0.1 mg/kg/day, IQR 0–0.1).

### Complete/partial renal response

Providers reported similar CR response rates for induction immunosuppression groups at 6 months; 46% of patients in the CYC group and 47% in the MMF group. Overall response (CR or PR) was reported in 83% (20/24) of patients in the CYC group vs. 59% (10/17) in the MMF group (*p* = 0.08). There was no significant difference between CYC and MMF and renal response (CR or PR) in multivariate logistic regression after controlling for age, gender, proliferative LN class, and steroid CTP.

Provider assessment of renal response (CR, PR) was confirmed by laboratory values in 24 of 41 (59%) patients. However, we were unable to corroborate the provider’s assessment in 17 patients due to: missing laboratory data (*n* = 9) and inconsistency between the laboratory values and the reported response (*n* = 8). To conservatively estimate the proportion of patients achieving renal response (CR or PR) at the 6-month visit using only the reported laboratory data, we counted the nine patients with missing data as non-responders, resulting in a CR rate just above 40% for both CYC (10/24, 42%) and MMF (7/17, 41%) groups. The total proportion of responders (CR or PR) in the CYC group was 63% (15/24) and 53% (9/17) in the MMF group, *p* = 0.54. Courses of non-responders are summarized in Additional file 2.

### Longitudinal outcomes: Proteinuria, GFR, SLEDAI-2 K

**Fig. 4:**
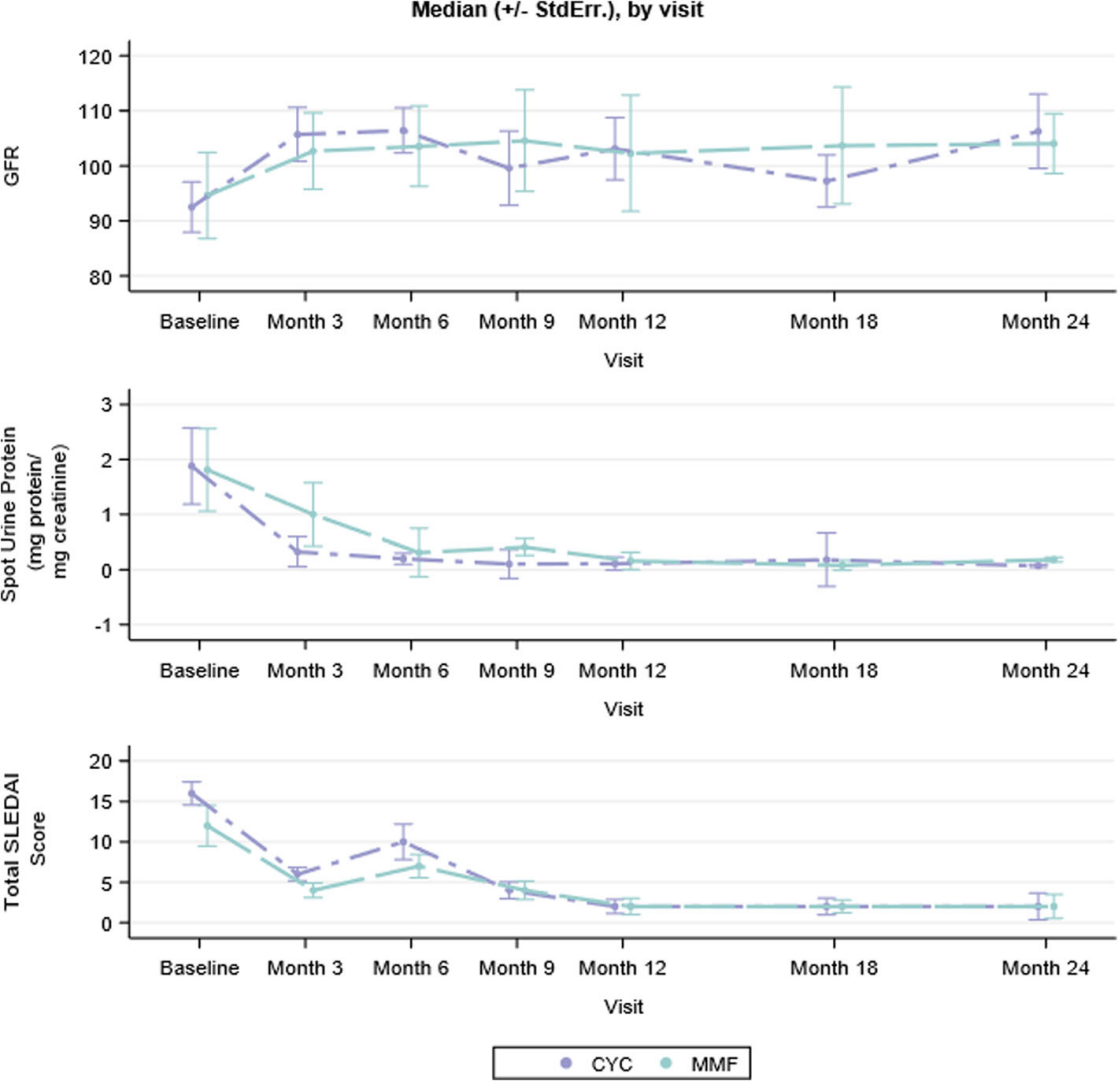
Estimated GFR, proteinuria, and SLEDAI over the study period by induction immunosuppression CTP. Abbreviations: CTP = consensus treatment plan, GFR = estimated glomerular filtration rate, SLEDAI = systemic lupus erythematosus disease activity index-2 K, StdErr = standard error

Median GFR, proteinuria, and SLEDAI-2 K scores over the course of the study are shown in Fig. 4. Exploratory analyses evaluating the effect of induction immunosuppression CTP (CYC vs. MMF) on outcomes of proteinuria, GFR, and SLEDAI-2 K over the study period were conducted using mixed effects models. No significant differences were found between CYC and MMF groups and GFR, proteinuria, or SLEDAI-2 K over time.

### Disease flares

Of the 30 patients with a CR or PR at month 6, four patients experienced disease flare (2 renal flares) by month 24; all four patients were on MMF at the time of flare.

### Adverse events

AEs are summarized in Table 4. Two serious adverse events were reported during the 6-month induction period; one patient was hospitalized for depression and suicidal ideation and one patient developed an opportunistic infection. Study is available in Additional file 3.

**Table 4:** Adverse Events

Patient	AE	Medications at time of AE	AE Grade	SAE	Timing
1	Depression with suicidal ideation	CYC, Mixed CS	3	Yes	Induction
2	Opportunistic infection	MMF, Mixed CS	2	Yes	Induction
3	Infusion reaction	CYC, Primarily IV CS	3	No	Induction
4	Steroid intolerance	MMF, Mixed CS	2	No	Induction
5	Hypertension	MMF, Mixed CS	3	Yes	Maintenance
6	Acute appendicitis	MMF	3	Yes	Maintenance
7	Chest pain	Mycophenolic acid, low-dose prednisone*	2	No	Maintenance
8	Gastroenteritis	MMF. low-dose prednisone*	2	No	Maintenance
9	Pyelonephritis	MMF, low-dose prednisone*	2	Yes	Maintenance

Abbreviations: *AE* adverse event, *CS* corticosteroid, *CYC* cyclophosphamide, *MMF* mycophenolate mofetil, *SAE* Serious adverse event

*Low-dose prednisone = 10 mg/day or less

## Discussion

Our pilot study illustrates the feasibility of adhering to the CARRA LN CTPs in clinical practice and collecting observational longitudinal data across ten US pediatric rheumatology centers. Most importantly, this study elucidates the need for revision of the steroid CTPs to reduce treatment variability and support future comparative effectiveness research as adherence to the steroid CTPs was poor (37% at 6 months) with large variability in dosing. The original CTP development process utilized case-based surveys to assess current practice of the CARRA membership and it is possible that the theoretical cases used to develop the CTPs did not allow for assessment of real-life nuance, treatment practices have changed, or the providers in this pilot study were not representative of those surveyed during the initial consensus process. Thus, as the current steroid CTPs do not seem to be representative of common use we recommend revision to include a faster tapering option for patients with early response to therapy or for those with dose-limiting steroid toxicity or intolerance.

We characterized physician decision-making and identified treatment biases that will be important to consider when designing future comparative effectiveness studies. Overall, the most common reason for selecting a particular CTP was “This is what I or my group always does” suggesting that although the aim was develop CTP options that were considered equivalent in effectiveness and would be equally acceptable as standard-of-care, providers may still have strong treatment preferences. Perhaps not surprisingly, there was a tendency to treat patients with class IV LN (vs. class III), hematuria, and compliance concerns with CYC. In addition, we observed increased use of the IV-based steroid CTPs (primarily IV and mixed) in the CYC group. While this is not surprising from a practical standpoint because it is more convenient for patients already receiving one IV medication to receive another, it may also reflect a tendency to treat patients with more severe disease and/or poor compliance with IV medications. Strategies to reduce the effects of confounding by indication such as cluster (site) randomization or statistical adjustment with propensity matching could be implemented in future CTP studies.

Another potential barrier to CTP implementation highlighted in this study will be developing a process to efficiently update CTPs as practice patterns evolve. Although 90% of pediatric rheumatologists surveyed during the CTP development process endorsed using CYC first-line for induction treatment of proliferative LN, the CYC CTP was used in 63% of patients [4]. In addition, the vast majority (93%) of patients received MMF for maintenance therapy. Taken together, these results likely reflect increased use of MMF by pediatric rheumatologists since development of the LN CTPs. In addition, the lack of AZA use for maintenance therapy is surprising given the comparable effectiveness to MMF demonstrated in adults [10–12], lower cost, and option for once daily dosing. During the LN CTP development process, the low-dose “Euro-lupus” IV CYC regimen was not included as an option because of the lack of dosing guidelines for children and because the CTPs are designed to reflect current practice and the Euro-lupus regimen was not commonly used by CARRA pediatric rheumatologists. In recent years, several U.S. pediatric rheumatology centers have begun using the Euro-lupus regimen in adolescents in light of data in adults demonstrating comparable long-term renal outcomes and lower risk of ovarian toxicity compared to conventional dosing [13, 14].

As the main objectives of this pilot study were to assess feasibility of adhering to the CTPs in clinical practice, the study was not powered to assess differences in clinical response between treatment groups. In an exploratory analysis estimating renal response using laboratory data and with patients with missing data as non-responders, both CYC and MMF groups had a CR rate just above 40% at the 6-month visit. Renal response criteria for LN are far from standardized however, when similar CR criteria (proteinuria < 500 mg/24 h, no worsening of GFR at 6 months) were applied to raw data sets from three large adult LN trials (Aspreva Lupus Management Study, Abatacept and Cyclophosphamide Combination Efficacy and Safety Study, and Euro-lupus Nephritis Trial), response rates for MMF, high-dose IV CYC, and low-dose IV CYC all groups showed CR rates of approximately 20%, substantially lower than our study [15–18]. Several factors that may contribute to this finding. First, this study included only new-onset proliferative LN patients while most adult proliferative LN trials do not exclude patients with prior LN flares and these patients may be less likely to achieve a CR. Second, many patients in the current study had their 6-month visit assessment late, leading to more time on therapy before outcome assessment, which could have favorably affected the response rate.

Our pilot study has several limitations. We were unable to corroborate provider assessment of renal response in many patients, most often due to missing laboratory values but there were also instances where the provider’s assessment did not match the reported laboratory data, raising concern regarding future use of provider response ascertainment. The inconsistency may indicate that the response criteria is difficult to apply in clinical practice and highlights the challenge of using research assessment tools designed for use in RCTs in the pragmatic study setting. Importantly, since this study was conducted, the CARRA registry has implemented measures to reduce missing data and improve data quality. In addition to known biases, this study is subject to bias from unmeasured confounders. Examples of potential unmeasured confounders in the current study are renal histopathology disease activity and chronicity and patient adherence. Baseline renal biopsy detail regarding activity and chronicity was not systematically collected but may have influenced provider decision-making regarding CTP selection. Poor medication adherence has been associated with poor renal outcomes in adults [19, 20] and although provider concern for poor adherence to oral medications was found to influence CTP selection, individual patient adherence data was not recorded and is difficult to measure. Lastly, given the small sample size, results from this feasibility study should be interpreted with caution.

## Conclusions

In summary, our pilot study demonstrates that the general approach of using the CARRA LN CTPs in clinical practice for observational research is feasible, however we identified several key issues to consider going forward, particularly revision of the steroid CTPs, determination of renal response, and strategies to reduce effects of confounding by indication.

## Additional files

Additional file 1: Concurrent medication use. (DOCX 12 kb)

Additional file 2: Course of Non-responders. (DOCX 11 kb)

Additional file 3: Weight gain. (DOCX 12 kb)

## Abbreviations

ACR: American College of Rheumatology
AE: Adverse event
CARRA: Childhood Arthritis and Rheumatology Research Alliance
CR: Complete response
CS: Corticosteroid
CTP: Consensus treatment plan
CYC: Cyclophosphamide
ESR: Erythrocyte sedimentation rate
GFR: Glomerular filtration rate
IQR: Interquartile range
IRB: Institutional review board
ISN/RPS: International Society of Nephrology-Renal Pathology Society
IV: Intravenous
LN: Lupus nephritis
MMF: Mycophenolate mofetil
PGA: Physician’s global assessment
PR: Partial response
SAE: Serious adverse event
SLEDAI-2 k: Systemic lupus erythematosus disease activity index-2000
Std Err: Standard error
UPCR: Urine protein to creatinine ratio
