# Supplementary material for: Pilot study comparing the childhood arthritis and rheumatology research alliance consensus treatment plans for induction therapy of juvenile proliferative lupus nephritis
Source: Pediatr Rheumatol Online J. 2018 Oct 22;16:65. doi: 10.1186/s12969-018-0279-0 (PMC6196456; doi:10.1186/s12969-018-0279-0)
Supplement: Supplementary file 1 — Concurrent medication use. (DOCX 12 kb) [file 12969_2018_279_MOESM1_ESM.docx]

# During the induction period, two patients were treated with additional immunosuppression, one patient in the CYC group received rituximab at month 5 and another was treated with tacrolimus in addition to MMF. No patients in the MMF group switched to mycophenolic acid (MyforticÒ) during the induction phase. All but one patient (98%) received hydroxychloroquine. Angiotensin converting enzyme inhibitor (ACE) or angiotensin receptor blocker (ARB) was prescribed in 45% of patients, this was more common in the CYC group (58%) compared to MMF (35%) though not a statistically significant difference (*p* = 0.208). Proton-pump inhibitor or histamine receptor-2 antagonists were used in 44% of patients. Supplemental calcium and vitamin D were used in 33% and 45% of patients respectively. Three of the 19 female patients in the CYC group received the GnRH agonist leuprolide. One patient was on contraception. Two patients in the CYC group received *Pneumocystis* prophylaxis with trimethoprim/sulfamethoxazole.
