# Supplementary material for: Pilot study comparing the childhood arthritis and rheumatology research alliance consensus treatment plans for induction therapy of juvenile proliferative lupus nephritis
Source: Pediatr Rheumatol Online J. 2018 Oct 22;16:65. doi: 10.1186/s12969-018-0279-0 (PMC6196456; doi:10.1186/s12969-018-0279-0)
Supplement: Supplementary file 2 — Course of Non-responders. (DOCX 11 kb) [file 12969_2018_279_MOESM2_ESM.docx]

There were 11 patients who did not achieve PR or CR per provider assessment at the 6-month visit. Of these 11, two met PR by laboratory criteria at 6 months and one was lost to follow-up after the 6-month visit. Of the remaining eight patients, four met response criteria by the 12-month visit. No patients required hemodialysis or kidney transplant.
