# Supplementary material for: Pilot study comparing the childhood arthritis and rheumatology research alliance consensus treatment plans for induction therapy of juvenile proliferative lupus nephritis
Source: Pediatr Rheumatol Online J. 2018 Oct 22;16:65. doi: 10.1186/s12969-018-0279-0 (PMC6196456; doi:10.1186/s12969-018-0279-0)
Supplement: Supplementary file 3 — Weight gain. (DOCX 12 kb) [file 12969_2018_279_MOESM3_ESM.docx]

Baseline absolute body mass index (BMI) was 22 kg/m2corresponding to a median BMI percentile of 81 (IQR 40–94). Just under half of patients (47%) met the Center for Disease Control criteria for overweight or obese. At the 6-month visit, the median absolute weight gain was 6 Kg, corresponding to a median BMI percentile 90 (61–97) and 63% of patients were overweight or obese. At the month 12 visit, the median BMI percentile was 93 (67–93) and 57% were overweight or obese. The association of steroid CTP with BMI change over time was estimated using a mixed model with repeated measures. Covariates included time of scheduled visits, baseline BMI, age, and gender. Only baseline BMI was significantly associated with follow-up BMI. Steroid CTP regimen was not significantly associated with follow-up BMI.
